# Effect of experimentally increased nutrient availability on the structure, metabolic activities, and potential microbial functions of a maritime Antarctic microbial mat

**DOI:** 10.3389/fmicb.2022.900158

**Published:** 2022-09-23

**Authors:** Antonio Camacho, Carlos Rochera, Antonio Picazo

**Affiliations:** Cavanilles Institute of Biodiversity and Evolutionary Biology, University of Valencia, Valencia, Spain

**Keywords:** microbial mats, Antarctica, inorganic nutrients (N and P), community structural and functional effects, metabarcoding and functional prediction

## Abstract

The role of competitive interactions based on resource utilisation was explored in a phototrophic microbial mat from Byers Peninsula (Maritime Antarctica). Shotgun metagenomic profiling of the mat showed a taxonomic and functionally diverse microbial community. The heterotrophic bacterial community was dominated by Proteobacteria, where genera typically found in polar habitats, such as *Janthinobacterium*, *Pseudomonas*, and *Polaromonas*, were highly prevalent. Cyanobacteria played the main role as primary producers, accompanied by diatoms and chlorophytes. To test the potential effects of the inorganic nutrient (N and P) availability on this community, a fully factorial nitrate and phosphorus addition experiment was conducted *in situ*. The mat exhibited a functional and structural response to the nutrient amendments. Compared to the undisturbed mat, phosphorus fertilisation favoured the growth of (non-heterocystous) cyanobacteria relative to that of diatoms, as indicated by changes in the carotenoid pigment biomarkers. Although no mat accretion was visible, fertilisation improved the phototrophic activity, and, mainly, when P was amended, the production of exopolymeric substances was favoured, whereas further changes in the vertical distribution of primary production activity were observed as well. Illumina amplicon sequencing of the 16S rRNA gene also demonstrated changes in the relative abundance of heterotrophic prokaryotes, which were detectable from the phylum to the genus level and mainly related to the amendment of nitrogen. Predictions made on the functional skills of these shifted prokaryotic communities indicated changes in abundance selecting taxa with a metabolic adaptation to the new nutrient scenarios. They mainly consisted of the enhancement of ecological strategies and metabolic regulatory mechanisms related to the uptake and metabolising of either nitrogen or phosphorus, regulated by its availability whether in a balanced way or not. This study is a pioneer in demonstrating how shifts in the regional dynamic of nutrients might alter the metabolic equilibrium of these initially considered homeostatic benthic communities. They can be accordingly considered as taxonomically diverse microbiomes with a functional repertoire still inclined to respond to the biogeochemical alteration of nutrient cycles, although occurring in a cold extreme environment where biological activity is partially restricted by environmental harshness.

## Introduction

Ecophysiological studies based on the manipulation of environmental variables, and the investigation of how these changes affect the structure and function of microbial communities, may help to interpret how direct and indirect effects of some components of global change, such as eutrophication and climate change, can affect these microbial communities in polar regions. Microbial mats, which are benthic multilayered communities that include heterotrophic and photoautotrophic microorganisms, are considered the appropriate models for studying interactions of biogeochemical fluxes and the role of microbes thereby (Camacho and de Wit, 2003; Bonilla et al., 2005; Stal and Noffke, 2011; Preisner et al., 2016). Several studies have addressed the effect of resource availability on microbial mats (e.g., Camacho and de Wit, 2003; Bonilla et al., 2005; Kohler et al., 2016). These studies revealed changes in the functional and structural features after the alteration of nutrient availability and their ratios, although the magnitude of these shifts varied among them.

Microbial mats are complex structures formed by multiple biofilms of microorganisms, essentially composed of prokaryotes and protists, embedded in a matrix of exopolysaccharides formed by their microbial components (Prieto-Barajas et al., 2018). Heterotrophic bacteria profit from the photosynthates of microbial primary producers, mainly cyanobacteria and microalgae. The Archaea and Eukarya can be also relevant in the formation and functioning of the microbial mats in different layers, although their abundance, community structure, and diversity can be highly variable between different types of microbial mats (Prieto-Barajas et al., 2018). There are multiple activities and metabolic processes that are crucial in the formation and maintenance of the mats (Bolhuis et al., 2014; De Anda et al., 2018) as those associated with nitrogen (nitrification, nitrogen fixation, and denitrification), phosphorus (alkaline phosphatase activity, low-affinity P-systems, etc.), and carbon metabolism (fuelling the heterotrophic way of life and others, such as those associated with exopolysaccharides production and consumption, or methanogenesis), as well as others related to environmental stress. Photosynthesis is the main source of energy for the microbial mat (Camacho and de Wit, 2003; Prieto-Barajas et al., 2018), where the top layer is typically dominated by oxygenic phototrophs, such as diatoms and cyanobacteria. The ecological success of benthic cyanobacteria, which are the main primary producers in terrestrial Antarctic environments, stands from the resistance to desiccation and high doses of ultraviolet radiation, as well as the absence of competition from planktonic autotrophs (Sabbe et al., 2004). These layered structures are abundant in the Byers Peninsula (Livingston Island and South Shetland Islands), and previous studies have demonstrated that they are, in part, dependent not only on moisture but also on nutrients availability (Fernández-Valiente et al., 2007; Velázquez et al., 2011; Rochera et al., 2013).

We hypothesised that, besides intense physical control, the microbial community structure of these mats can be partially regulated by biotic interactions, such as resource competition or others. In fact, the potential occurrence of strong biotic interactions, even cascading effects due to predation, was demonstrated a long time ago for lakes of the Byers Peninsula under the milder summer conditions in maritime Antarctica (Camacho, 2006). Attending to mechanisms described by the resource competition theory (Tilman, 1982; Tilman et al., 1986), it is expected that an unbalanced availability of nutrients induces a shift in the relative dominance and the activity of different organisms, and these changes affect the whole microbial mat. For instance, the relevance of certain bacteria, such as those of the genus *Pseudomonas*, in the structure and function of microbial mats, especially regarding the exopolysaccharides matrix that binds the mats, has been widely studied (e.g., Mukherjee et al., 2022). Other groups of heterotrophic bacteria can also be very relevant, not only in the biosynthesis and recycling of exopolysaccharides but also in key metabolisms for mat structure, including associations with the photosynthetic fraction (Brambilla et al., 2001; Valdespino-Castillo et al., 2018; Jackson et al., 2021). On the contrary, cyanobacteria and eukaryotic microalgae, which dominate the photosynthetic fraction of the Byers microbial mats (Fernández-Valiente et al., 2007; Rochera et al., 2013; Almela et al., 2019), may have different efficiencies to take up nutrients. Some cyanobacteria, for instance, can take a competitive advantage if they are capable to obtain nitrogen from N_2_ fixation or from storage compounds (e.g., cyanophycin, Watzer and Forchhammer, 2018). Additionally, some cyanobacteria can accumulate inorganic phosphorus as polyphosphates (Sanz-Luque et al., 2020).

We experimentally tested these ideas by studying the effects of artificially increasing the concentration of inorganic N, P, and both in a polar microbial mat. Among the diverse typologies of the microbial mat occurring in the Byers Peninsula, we chose the Stream Mat described by Fernández-Valiente et al. (2007) displaying high moisture. Experiments were performed during the Antarctic summer with an average air temperature of several degrees over the water freezing point, attempting to avoid the strong physical control that may disguise other underlying biotic and abiotic regulatory factors. After the addition of nutrients, changes in the main structural and functional characteristics of the mat were assessed by a multi-proxy approach. Besides low temperatures and the Antarctic isolation factor, the low nutrient status may strongly influence the biotic and functional diversity of Antarctic systems (Rochera and Camacho, 2019). Accordingly, in this study, our objective was to determine the potential changes in the community structure and the main functional traits that may be promoted in an Antarctic microbial mat after the experimental alteration of nutrient conditions. With that, we aimed to give insights into how the activation of biogeochemical cycles by climate change, which will increase the transit of nutrients in polar regions (Camacho et al., 2012), could influence these key ecosystems for polar areas.

## Materials and methods

### Study site

The studied microbial mat was located in the water-flooded shore of a small stream located at South Beaches of the Byers Peninsula (62° 38′ S, 61° 01′ W), Livingston Island (South Shetland Islands), Antarctica. The Byers Peninsula is a 60.6 km^2^ area mostly free of ice and snow cover during part of the Antarctic summer (Toro et al., 2007). In the studied season, most summer ice thaw in Byers occurred in late December and early January, and then water flowed through a drainage network of small and short streams until the next frozen season. Usual weather conditions in January were overcast as about 60% of the period with rain (mostly) or snow (seldom) for 30% of the time. January monthly rainfall was 54 l/m^2^, with an average temperature of 2.2°C. The studied microbial mat is permanently covered by water, but water velocity in the overlying waters was very low, since most flows circulated through the central part of the stream channel, which was not covered by this mat. The experimentally studied mat corresponded to the Stream Mat described by Fernández-Valiente et al. (2007). All the necessary permissions were granted by the Spanish Polar Committee for the work performed in Byers Peninsula during the period of experiments. Byers Peninsula has a special protection regime as being an Antarctic Special Protected Area (ASPA No. 126).

### Water physical and chemical analyses

Dissolved oxygen, conductivity, and pH were measured *in situ* with a YSI (YSI Inc., Yellow Springs, OH, USA) multiprobe. Before incubations, the concentrations of dissolved inorganic compounds of nitrogen, phosphorus, and silica were determined in both the overlying water and the interstitial water of the mat, following standard analytical methods (APHA-AWWA-WPCF, 2005). Interstitial water was obtained by applying pressure on untreated fresh mat cores, thus allowing that pore water came out from the mat. Once incubations were finished, the overlaying water of each microcosm was sampled again to measure variations resulting from the experimental nutrient enrichment. All samples for nutrient analyses were filtered *in situ* through GF/F glass fibre filters and kept frozen until analysis. Nitrate plus nitrite (NO*_*x*_*) was measured after reduction of nitrate to nitrite and then by determination of nitrite with sulfanilamide and *N*-(Naphtyl-1)-ethylenediamine dichlorhydrate (NNED) (method 4500-NO3-E; APHA-AWWA-WPCF, 2005). Ammonia was analysed using the phenol-hypochlorite method (Golterman, 2004). Soluble reactive phosphorus was determined by the phosphomolybdic acid-ascorbic method (method 4500-P-E; APHA-AWWA-WPCF, 2005) and soluble reactive silica by the molybdosilicate method (method 4500-SiO2-C; APHA-AWWA-WPCF, 2005). Total dissolved nitrogen (TDN) was quantified from filtered samples by oxidation with alkaline persulphate digestion at 150°C for 2 h. After neutralisation of samples, nitrogen present in samples was quantified following the same method described for nitrate. The sum of NO*_*x*_* and NH_4_ was considered as total dissolved inorganic nitrogen (DIN), and dissolved organic nitrogen (DON) was obtained by subtracting DIN from the total dissolved nitrogen (TDN). All nutrient determinations were made spectrophotometrically using a Beckman DU-7 spectrophotometer.

### Mat sampling and experimental setting

**FIGURE 1:**
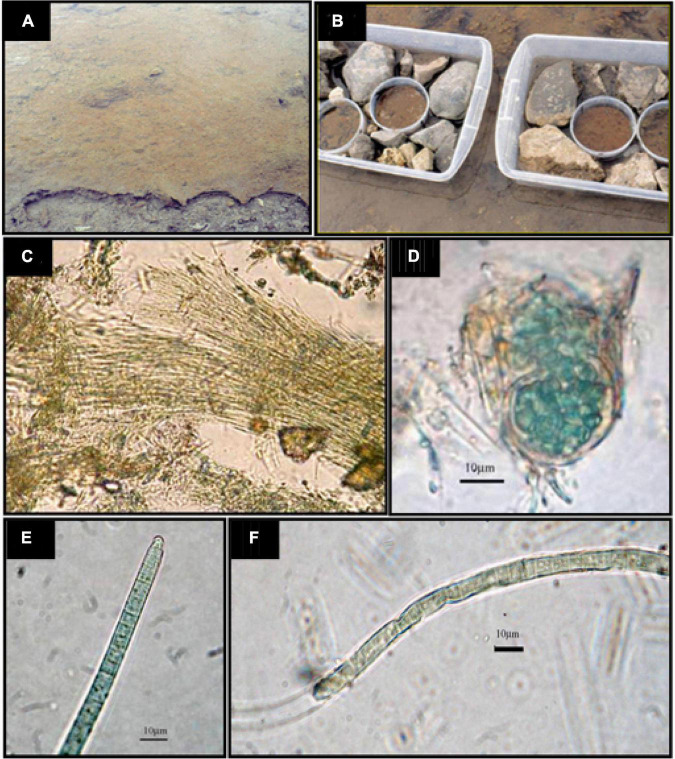
**(A)** An image of the site where samples were picked up. **(B)** The fragments of the mat for experimental nutrient additions were disposed of into PVC containers with a surface of 70 cm^2^ and then submerged in stream water supplemented with nutrients (except the controls) per triplicate, according to the experimental design. Samples were prepared, taking care that fragments were perfectly fitted in with the container walls. **(C)** *Leptolyngbya* sp.; **(D)** *Chroococcidiopsis* sp.; **(E)** *Phormidium autumnale*; **(F)** *Oscillatoria* sp.

Microbial mat samples of a depth of 6 cm were collected from the studied site (Figure 1A), placed in 70-cm^2^ plastic containers (Figure 1B), and located again in the place of origin in order to maintain natural temperature and illumination conditions during the incubation. Then, they were covered with the stream water (filtered through a GF/F glass fibre filter), usually covering the mat (Figure 1A). These microcosms were amended, when convenient, to establish the following incubation conditions: (1) C – “Control”, without nutrient additions; (2) +N – amended with NH_4_NO_3_ to a final concentration of 60 μmol N l^–1^; (3) + P – amended with NaH_2_PO_4_ to a final concentration of 4 μmol P l^–1^; and (4) + NP – amended with NH_4_NO_3_ to a final concentration of 60 μmol N l^–1^ and NaH_2_PO_4_ to a final concentration of 4 μmol P l^–1^. The + NP additions show an N/P molar ratio of 15, that is, according to the Redfield ratio, which is optimal for benthic microalgae (Hillebrand and Sommer, 1999). Four replicates were assayed for each of the treatments. Incubations were conducted for 16 days to determine the effect of the nutrient amendment on the community structure and function. Nutrient amendments were performed both at the beginning of incubations and on Day 8 to reach the final concentrations mentioned previously. The water temperature during the incubation period ranged between 1.64 and 2.77°C. Considering the low-growth rates of these microbial communities, the experiment was run during this time for detecting changes, if any, within the microbial community of the mat. Possible changes in the abundance of the different groups of photosynthetic microorganisms of the mat (diatoms and cyanobacteria) were studied by means of the chemotaxonomy of taxon-specific photosynthetic pigments (Camacho and de Wit, 2003) and metabarcoding. Shifts in the taxonomic functional structure of the prokaryotic community were assessed by 16S rRNA gene amplicon sequencing.

Additionally, the possible effect of the nutrient addition on inorganic ^13^C-carbon assimilation, depth-specific oxygenic photosynthesis (measured with microelectrodes), inorganic nitrogen (ammonium and nitrate) uptake, N_2_ fixation (acetylene reduction), total carbon, total nitrogen, and total phosphorus accumulation, as well as the percent of organic matter in the sediment, was also studied as described below.

### Bulk biomass, elemental composition, and extracellular polymeric substances

The percent of organic matter in the sediment, as well as total carbon (TC), total nitrogen (TN), and total phosphorus (TP) relative (w/w, and then transformed to molar ratios) contents, was determined in sediment cores of 700 mm^2^ of a surface and 5 mm in depth collected at the end of the experiment. Dry weight was determined after drying at 103°C for 6 h, and the weight of the organic matter was determined after the ignition of dry samples at 460°C (APHA-AWWA-WPCF, 2005). TC and TN were determined with an elemental CHNS analyser (Carlo-Erba EA-1110). TP was determined after persulphatic-acid hydrolysis of the mat at 135°C for 2 h to hydrolyse any P-form to orthophosphate, and then this was measured as previously described for soluble reactive phosphorus after pH neutralisation.

The amount of extracellular polymeric substances (EPS) in the experimentally studied microbial mats was assessed separately for their main components (carbohydrates and proteins). For this analysis, a known weight from lyophilised samples was placed in 1.5-ml centrifuge tubes, and 1 ml of 2% EDTA was added; subsequently, the tubes were stirred and heated at 35°C for 1 h. Then, they were centrifuged at 10,000 rpm for 10 min, and the supernatant was recovered for analysis. The carbohydrate content of the extract was obtained by measuring the hexose equivalent (polysaccharide) content in the sample by the phenol-sulphuric acid spectrophotometric method (Herbert et al., 1971) using glucose as the standard. The protein amount was quantified according to Bradford (1976), taking bovine serum albumin (BSA) as the standard.

### Microscopic and photosynthetic pigments study

Mat samples were fixed with 4% formaldehyde and examined using a light microscope (Nikon Eclipse E600, with a Canon Power Shot G6 Digital Camera). Morphological identification of cyanobacterial species was performed by identification of size morphotypes suggested by Broady and Kibblewhite (1991).

Sediment cores of mat samples used for pigment concentration determination in the different experimental conditions were collected at the end of incubations and stored frozen at −20°C during the Antarctic expedition and further at −80°C until being analysed. Photosynthetic pigments were extracted with 90% acetone (chromatography grade) at 4°C using three successive extractions, after sonication, of 1, 6, and 12 h, respectively. The three sequential extracts were then pooled together and evaporated by vacuum. Later, they were redissolved in a smaller volume of acetone and filtered previously to the injection in the chromatographic system. Separation was performed with a Waters 2690 HPLC system equipped with a photo-diode array detector (Waters 996) on a Spherisorb S5 ODS2 chromatography column, following the procedure described by Picazo et al. (2013). Peaks were identified according to their absorption spectra, and pigment concentrations were estimated using standards prepared with commercially available purified pigments (DHI, Denmark). Chlorophyll *a* (Chl-*a*) concentration was considered a proxy for the total biomass of phototrophs. An indication of the health status of the phototrophic community was obtained by using the ratio between Chl-*a* and its major degradation product (i.e., Pheophytin *a*) (Camacho and de Wit, 2003). Myxoxanthophyll and fucoxanthin were determined as taxa-specific carotenoids for quantification of the relative abundance of cyanobacteria and diatoms, respectively, and possible changes in the relative abundance of these two groups associated with the treatments were deduced from differences in the fucoxanthin/myxoxanthophyll molar ratio among treatments at the end of the incubations.

### DNA extraction, metagenomic analyses, and 16S rRNA sequencing

Two methodological approaches were used for profiling microbial communities: (i) the metagenomic shotgun for characterising the original mat and (ii) 16S rRNA gene amplicon sequencing for assessing the effects of the nutrient-amended treatments compared to the control with no additions. The former was conducted with only a replicate of the control treatment (C) to provide the broadest characterisation of the microbial community, which included bacteria, archaea, viruses, eukaryotes, and fungi. On the contrary, the most cost-effective procedure of sequencing 16S rRNA gene amplicons was performed with the three replicates of the C treatment and with a replicate of each of the nutrient-amended treatments (+N, +P, and +NP). This was made to identify the major effects of nutrient enrichment on the prokaryotic community. This provided information on the taxonomic structure of the community, being also possible to report metabolic functional information based on taxa abundances using the predictive algorithm PICRUSt2 (Douglas et al., 2020). We used complementarily both the shotgun and amplicons sequencing, seeing that broad-scale diversity patterns show to be largely consistent when both methods are compared (Rausch et al., 2019). By sequencing the control treatment (C) simultaneously with the two methods, it would be possible to detect potential biases between the methods whenever they occur.

For all sequencing procedures, DNA extraction from microbial mats was performed with the EZNA Soil DNA isolation kit (Omega Bio-Tek, Inc., Norcross, GA, United States), following instructions of the supplier, and extracts were subsequently quantified with Nanodrop as described by Picazo et al. (2019). Shotgun sequencing of Sample C was performed by the Illumina Novaseq6000 150PE (150 bp × 2 bp) (Macrogen), which provided ca. 120 million clean reads and 15 Gb of output. For the three replicates of the control treatment (C) and one replicate of nutrient-amended treatments (+N, +P, and +NP), the sequencing of the region V4 of the 16S rRNA gene was done using the Illumina MiSeq system (2 bp × 250 bp). For each sample, Illumina-compatible, dual-indexed amplicon libraries of the 16S-V4 rRNA hypervariable region were created with primers 515f/806r. PCR reactions were made following Kozich et al. (2013). Completed libraries were batch normalised using Invitrogen SequalPrep DNA Normalisation Plates. Then, the Qubit quantified pool was loaded on a standard Illumina MiSeq v2 flow cell, and sequencing was performed in a 2 bp × 250 bp paired-end format using a MiSeq v2 500 cycle reagent cartridge. Custom sequencing and index primers complementary to the 515/806 target sequences were added to appropriate wells of the reagent cartridge. Base calling was done by Illumina Real Time Analysis (RTA) v1.18.54, and the output of RTA was demultiplexed and converted to FastQ format with Illumina Bcl2fastq v2.19.1.

### Taxonomic annotation, clustering, and functional analysis

The taxonomic annotation of one control (C) sample metagenome was performed using the metagenome classifier Kaiju (Menzel et al., 2016), which uses the more evolutionarily conserved amino acid sequence to perform a comparison with the NCBI BLAST nr database. The Kaiju source code and the web server are available at http://kaiju.binf.ku.dk. Taxonomic annotation was performed with the Pavian library in R (R Development Core Team, 2011; McMurdie and Holmes, 2013). The taxonomic annotation data were used to build stacked plots at the domain, phylum, and genus levels.

Amplicon 16S rRNA (V4) sequences were processed using the UPARSE pipeline using USEARCH v11.0.667 (Edgar, 2013). After merging of read pairs, the dataset was filtered by a maximum number of expected errors of 0.5. Chimeric sequences were removed with USEARCH v11.0.667, utilising the UCHIME (Edgar, 2013), against the SILVA 138.1 database. Filtered sequences were clustered in zero-radius Operational Taxonomic Units (ZOTUs), which are sequences with 100% identity. Alignment and taxonomic assignment were done with SINA v1.2.1152 using the SILVA 138.1 database (Pruesse et al., 2012). SINA uses the Lowest Common Ancestor method (LCA). We configured a “Min identity” of 0.8, and a maximum number of search results of 1 per sequence results in a “best match” type. Sequences with low alignment quality (<75%) and sequences identified as mitochondria or chloroplasts were removed from the analysis. The original ZOTU table was normalised by rarefying the reads of all samples to the minimum threshold of 9,130 reads/sample. Rarefactions were repeated 100 times to avoid the loss of less-abundant ZOTUs, and then, rarefactions were unified in three average rarefied ZOTU tables.

Functional predictions of metagenome in the microbial community were inferred using the Phylogenetic Investigation of Communities by Reconstruction of Unobserved States (PICRUSt2) from the 16S rDNA gene data, as described by Douglas et al. (2020). These functional predictions were made assuming that phylogenetically closely related taxa identified by the 16S rRNA gene share a higher degree of features than distantly related taxa. The analysis was conducted with the raw ZOTUs table (i.e., previously to the filtering and rarefaction steps) after performing the normalisation with the PICRUSt2 tool. The accuracy of functional predictions was tested through the Nearest Sequenced Taxon Index (NSTI), which measures the average distance between OTUs and their nearest sequenced genome representatives available to PICRUSt2. The NSTI scores assess the robustness of PICRUSt2 predictions by assessing the availability of reference genomes closely related with the sequence of the sample. Thus, higher NSTI scores indicate a low availability of related references in such a way that functional predictions may result in less accuracy. By contrast, lower scores occur when the availability of reference genomes is adequate to provide predictions. Following general criteria (Douglas et al., 2020), sequences with an NTSI higher than two were discarded for the analysis. The average NTSI of sequences obtained in this analysis was then 0.12. Functional inferences of the prokaryotic community were then performed by screening the available annotated genes within the Kyoto Encyclopedia of Genes and Genomes (KEGG^1^) catalogue, following Ye and Doak (2009).

### Sequencing result deposition

All sequence data from this study have been deposited in the Sequence Read Archive (SRA) of the National Center for Biotechnology Information (NCBI), BioProject accession No. PRJNA817827.

### Microelectrode studies on light-saturated photosynthetic activity

The rates of oxygenic photosynthesis were estimated for each of the replicates of the different incubation conditions at depth intervals of 200 μm through the sediment core by measuring the decline in oxygen concentration (the slope of the curve) after shifting the samples from light-to-dark conditions for 4 s (Camacho and de Wit, 2003). Oxygen concentrations were determined with a polarographic Clark-style oxygen microelectrode (Diamond General, Ann Harbor, MI, United States), while mat samples were exposed to constant saturating illumination of 750-μmol photons m^–2^ s^–1^ given by a halogen lamp. The mean temperature of the overlying water during measurements was 6.6°C, and the pH was 7.64. Photosynthetic rates were corrected for sediment porosity and integrated over the sediment column to calculate areal rates. Two profiles of photosynthetic activity were made at different spots in each of the three replicates for each of the four different conditions assayed, and mean values and standard deviations are shown in the respective figures. All microelectrode measurements were confined to the active layer of the mat (i.e., 0–3 mm). With the results obtained, both oxygen production and consumption were also estimated by observing the oxygen fluxes against depth. Fluxes were calculated according to Fick’s first law of diffusion (Cohen and Rosenberg, 1989) using the following equation:


(1) $$J_{z}=\phi \,D_{s}\,\left(d\,C/d\,x\right)$$

where *Jz* is the flux through the layer at depth *z*, Φ is the porosity of a mat, *Ds* is the diffusion coefficient of oxygen within the mat, and *dC/dx* is the slope of the oxygen profile against deepness at depth *x*. A negative flux refers to a net downward flux, whereas a positive flux refers to a net upward flux.

### Inorganic carbon assimilation in the mat

Inorganic carbon assimilation in the mat was measured by the stable isotope technique (^13^C uptake). Three sediment cores of 700 mm^2^ of a surface and 5 mm in depth were obtained at the end of the incubation period from each of the different treatments and replicates after microelectrode measurements. Each sediment core (per triplicate) was placed in a hermetic plastic Whirl-pak^®^ bag (3 cores per bag), containing 10 ml of stream mat-overlying water filtered through a GF/F glass fibre filter, and 100 μl of a stock solution 1-g C l^–1^ of NaH^13^CO_3_ (99% of ^13^C atoms) was added. The natural concentration of dissolved inorganic carbon (DIC) availability was determined by measuring total alkalinity (APHA-AWWA-WPCF, 2005), and then calculated by considering the chemical DIC equilibrium according to pH and temperature values. Incubations were conducted for 3 h by locating these plastic bags in the stream to maintain natural temperature and illumination conditions, placing the cores with the surface layer facing up, and avoiding overlapping. Four incubation conditions were assayed for each sample as follows: (a) under ambient irradiance; (b) under ambient irradiance and supplemented with 3-[3,4-dichlorophenyl]-1,1-dimethylurea] (DCMU) to a final concentration of 10^–5^ M to eventually measure anoxygenic photosynthesis (DCMU specifically inhibits photosystem II in oxygenic autotrophs); (c) incubation in the dark without further additions; and (d) incubation in the dark with the addition of formaldehyde to a final concentration of 4% (to assess the passive non-biological deposition of labeled carbon by chemical precipitation). All these incubation conditions allowed us to distinguish between oxygenic photosynthesis and possible anoxygenic photosynthesis, dark inorganic-C fixation (chemolithotrophy), and passive ^13^C-carbonate precipitation, if any. The mean temperature of overlying waters during measurements was 3.3°C and pH 7.62, whereas irradiance during incubation was 589 ± 224-μmol photons m^–2^ s^–1^. Incubations were stopped by adding 3-ml HCl 1N, and samples were aerated for 3 h to allow the non-fixed NaH^13^CO_3_, escaping in form of ^13^CO_2_. After that, the samples were neutralised, adding 3-ml NaOH 1N, for 1 h, and then the liquid was poured from the bags, retaining the cores, which were quickly washed with GF/F-filtered stream water. During transportation, cores were stored frozen at −20°C in darkness. Once in the laboratory, the cores were dried at 60°C and grounded by a pestle and a mortar for further analyses. The natural abundance of ^13^C and isotopic enrichment in the assayed samples were measured with an IRMS Micromass-Isochrom mass spectrometer and calculated following Alcamán-Arias et al. (2018). In addition to the experimental set, inorganic carbon photoassimilation was also measured five more times in an undisturbed mat during January.

### Nitrogen assimilation in the mat

The uptake of N from ammonium and nitrate was measured using the ^15^N stable isotope technique. Three sediment cores of 700 mm^2^ of a surface and 5 mm in depth were obtained at the end of the incubation period from each of the different treatments and replicates. Cores were placed in Whirl-pak^®^ bags (three cores per bag), containing 10 ml of GF/F-filtered stream mat-overlying water. To set up the assay, 50 μl of stock solutions (1-mg N l^–1^) of N-NH_4_^+^ or N-NO_3_^–^ was added as (^15^NH_4_)_2_SO_4_ (98% of ^15^N atoms) or as K^15^NO_3_ (99.9% of ^15^N atoms), depending on experiments. Triplicate samples were incubated for 3 h at the stream shore to maintain natural temperature and illumination conditions (as well as in the dark), avoiding the core overlapping within each bag. The mean temperature of the overlying water during measurements was 2.8°C and pH 7.63; irradiance during incubation was 346 ± 55-μmol photons m^–2^ s^–1^. After incubation, the samples were rinsed three times with filtered water to eliminate unincorporated ^15^N, and then they were frozen at −20°C during the transport to the laboratory at the end of the field expedition. Once in the laboratory, the cores were dried at 60°C and grounded by a pestle and a mortar. Additional cores were taken for the determination of ^15^N natural abundance. The natural abundance of ^15^N and isotopic enrichment of the assayed samples were measured with an IRMS Micromass-Isochrom mass spectrometer and calculated following Alcamán-Arias et al. (2018).

Nitrogenase activity was measured by the acetylene reduction technique (Fernández-Valiente et al., 2007). Mat cores from the experimental settings were incubated in triplicate in 275-ml plastic flat bottles (three cores per bottle), containing 60 ml of filtered water taken from the stream at the study site. Plastic bottles were capped with reversible rubber stoppers and parafilm to assure gas tightness. The same volume of acetylene was added to replace 10% of the air, and incubations were conducted for 4 h at the shores of the stream to maintain natural temperature and illumination conditions. Special care was taken to place the cores with the surface layer facing up and to avoid overlapping. At initial and final incubation times, the gas samples were collected with double needles in 10-ml pre-evacuated Vacutainer tubes, which were brought to the lab at the end of the field campaign. The mean temperature of the overlying water during measurements was 2.9°C and pH 7.64; irradiance during incubation was 352 ± 57-μmol photons m^–2^ s^–1^. The ethylene concentration was then determined after incubations in duplicate for each sample, using a gas chromatograph (Shimadzu model GC-8A), equipped with a flame ionisation detector using a Porapak N 80/100 column.

### Statistical analyses

To compare the results of the different measured variables for each of the treatments and the controls, a one-way ANOVA was used. The Levene test for variance homogeneity was performed prior to using parametric non-parametric analyses made when the test was not satisfied. *A posteriori* multiple comparisons of the means were made using the Duncan test. Some of the parameters (all except photosynthetic activity, TOC, and TN) were also determined before incubation for at least one of the different replicates for each treatment; no significant differences (*p* < 0.05) among replicates were detected. Differences at the end of the experiment were thus attributed to treatment effects (control vs. nutrient additions).

Two distance-based redundancy analyses (dbRDA) were performed to describe the ordination of the main taxa and metabolic predictions (selected genes) in an environmentally constrained space, respectively. The relevance of the metabolic processes was defined based on the PICRUSt2 predictions from genes associated with the metabolism of major nutrients, stress, autotrophy, and exopolysaccharide biosynthesis (Supplementary Table 1). The analyses were conducted with the vegan R package (Oksanen et al., 2017). A total of 14 variables (C, carbon; N, Nitrogen; P, Phosphorus; EPS carb, Carbohydrate exopolymers; EPS prot, Protein exopolymers; chla, Chlorophyll *a*; Myxo, Myxoxanthophyll; Fuco, Fucoxanthin; Lut, Lutein; Pheo, phaeophytin; C uptake, NO_3_ uptake, NH_4_ uptake, and Nit.act, Nitrogenase activity) were used as explanatory variables. The values of these variables were the average observed for each of them in the four experimental treatments (C, +N, +P, and +NP). Data were square root transformed and normalised before building a Euclidean resemblance matrix.

## Results

### Environmental variables and water chemical conditions

Typical summer in Byers Peninsula is cloudy, rainy, and windy, and temperatures are usually over 0°C, although the weather conditions are known to be highly variable. During the incubation, performed in January, most days followed this pattern, with only 2 sunny days (irradiance at noon c.a. 1,000 μE m^–2^ s^–1^). Thus, most times, it was cloudy (irradiance at noon c.a. 350 μE m^–2^ s^–1^), and the day temperature typically oscillated from −2 to 4°C on these cloudy days. The stream water overlying the microbial mat at the time of sampling had a low salt content (78 μS cm^–1^) and low concentrations of inorganic nitrogen (nitrate and ammonia) and soluble phosphorus (SRP); however, silica concentrations were enough high to preclude any silica limitation for diatoms (Table 1). In comparison, mat pore waters contained higher ammonia soluble and phosphorus concentrations than overlying water (more than triple). By considering these mat pore water concentrations, nutrient addition increased actual concentrations by five times for nitrogen and by two times for phosphorus. At the end of incubations, nutrient concentrations increased in the overlying water accordingly with the nutrient amendment. Almost nutrient exhaustion (especially ammonia and SRP) occurred in the non-amended controls, whereas SRP exhaustion occurred in the N-amended treatment (when only N was added), and comparable ammonia and nitrate exhaustion occurred in the P-amended microcosms, whereas final nutrient concentrations in the overlying waters of the N + P treatments were high, showing nutrient saturation (data not shown). Among nitrogen sources, after incubation, ammonium dropped to lower concentrations, showing that it was the preferred form for inorganic nitrogen uptake by mat microorganisms. Treatments supplemented with nitrogen showed higher concentrations of total dissolved nitrogen in the overlying water, but these nitrogen pools were mainly composed of nitrate and dissolved organic nitrogen (DON), with ammonium being the scarcest fraction. On the contrary, in the controls and treatments amended with phosphorus, the DON was clearly the main nitrogen form, whereas ammonium was depleted.

**TABLE 1:** Molar concentrations (μmol L^–1^) of main inorganic nutrients measured in the overlaying and interstitial water of the microbial mat at the beginning of the experiment.

Nutrient	Overlaying water	Interstitial water
NH_4_	3.22	11.2
NO_x_	1.71	1.93
SRP	0.487	1.6
SRSi	77.2	37.2

Ammonium (NH_4_), nitrate plus nitrite (NO_x_), soluble reactive phosphorus (SRP), and soluble reactive silicate (SRSi).

### Morphological description of the cyanobacterial assemblages

Due to the morphological simplicity of some organisms and the difficulties to observe thick mats, only a few genera were reliably identified. The mat samples were all dominated by the thin, filamentous, non-heterocystous cyanobacterium *Leptolyngbya*, with trichomes being 1 to 1.2μm wide, embedded in a gelatinous mucilage, determined the mat structure (Figure 1). The top layer of the mat also included diatoms and other eukaryotic microalgae, whereas filamentous cyanobacteria were mostly abundant in deeper layers. Several other cyanobacterial morphotypes, mainly non-heterocystous and filamentous cyanobacterial taxa, co-occurred with *Leptolyngbya*, mostly belonging to the order Oscillatoriales (Figure 1). When the size morphotypes suggested by Broady and Kibblewhite (1991) were used, most of the cells were representative of morphotypes B (diameter, 3.6–5.4 μm) or D (diameter, 6.9–8.2 μm). Unicellular cells, aggregated and enclosed in a common yellow sheath, were also observed and described as *Chroococcidiopsis* sp. No filamentous heterocystous taxa were observed in any of the enriched mat treatments or in the controls without nutrient amendments.

### Metagenomic description of the microbial community in the undisturbed mat

**FIGURE 2:**
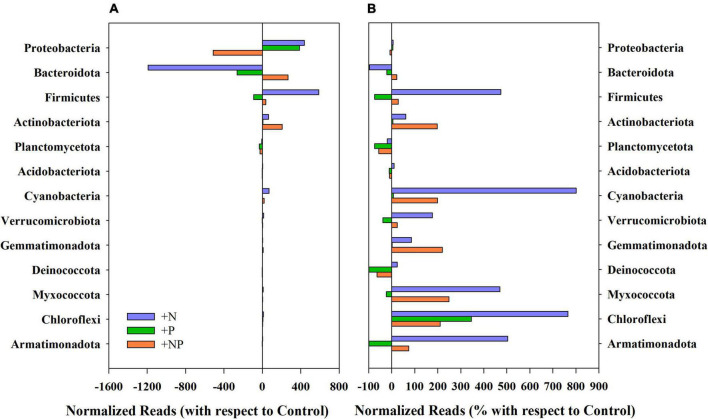
**(A)** Variation of the normalised reads in the different nutrient amendment treatments with respect to the control for the main phyla present in the studied microbial mat. **(B)** Percentage of variation of the normalised reads in the different nutrient amendment treatments with respect to the control for the main phyla present in the studied microbial mat.

Shotgun analysis performed with the undisturbed mat provided a total of 945,236 sequence reads per gigabase (reads/Gb). Most of the reads belonged to Eubacteria and were allocated in 31 phyla (Figure 2), with the more abundant Proteobacteria (91.5%), Bacteroidetes (4.7%), Actinobacteria (1.5%), Planctomycetes (1.4%), Firmicutes (0.5%), and Verrucomicrobia (0.2%). Proteobacteria being mainly composed of typical genera from polar environments, such as *Janthinobacterium*, *Pseudomonas*, and *Polaromonas*, whereas Bacteroidetes reads mainly belonged to the genus *Pedobacter*. Archaea also occurred in the mat, although with a notable lower abundance (742 reads/Gb), compared to Eubacteria. The chemolithoautotrophic nitrifier *Nitrosopumilus* and diverse methanogens (i.e., *Methanothrix*, *Methanosarcina*, and *Methanocaldococcus*) were the most important functional groups in the archeome.

The metagenomic analysis revealed a phototrophic community principally composed of cyanobacteria (3,756 reads/Gb), chlorophytes (31 reads/Gb), and diatoms (8 reads/Gb). Cyanobacterial reads, whose dominance coincided with that observed microscopically, were mainly assigned to genera *Leptolyngbya* (35%), *Phormidium* (13%), *Pseudanabaena* (7%), and *Nostoc* (4%), although other filamentous genera, such as *Microcoleus*, *Oscillatoria*, and *Calothrix*, were also detected. The less-abundant unicellular forms were mainly represented by *Synechococcus*, *Cyanobium*, and the colonial *Chroococcidiopsis*. Among the diatoms, the reads were primarily assigned to the genera *Thalassiosira* (36%), *Fragilariopsis* (24%), *Nitzschia* (12%), *Phaeodactylum* (10%), *Pseudo-nitzschia* (6%), *Fistulifera* (5%), and *Eunotia* (2%). Although scarcer, the chlorophytes were also diverse, with 33 identified genera, including unicellular and colonial forms of genera *Chlorella* (16%), *Coccomyxa* (9%), *Chlamydomonas* (7%), *Scenedesmus* (7%), *Micromonas* (6%), *Gonium* (5%), *Dunaliella* (5%), *Pycnococcus* (5%), and *Volvox* (4%). Some reads of flagellated cryptomonads such as *Guillardia* or small Prymnesiophytes such as *Chrysochromulina* were also recognised in the mat.

### Structural changes in the prokaryotic community by 16s rRNA amplicon sequencing

**FIGURE 3:**
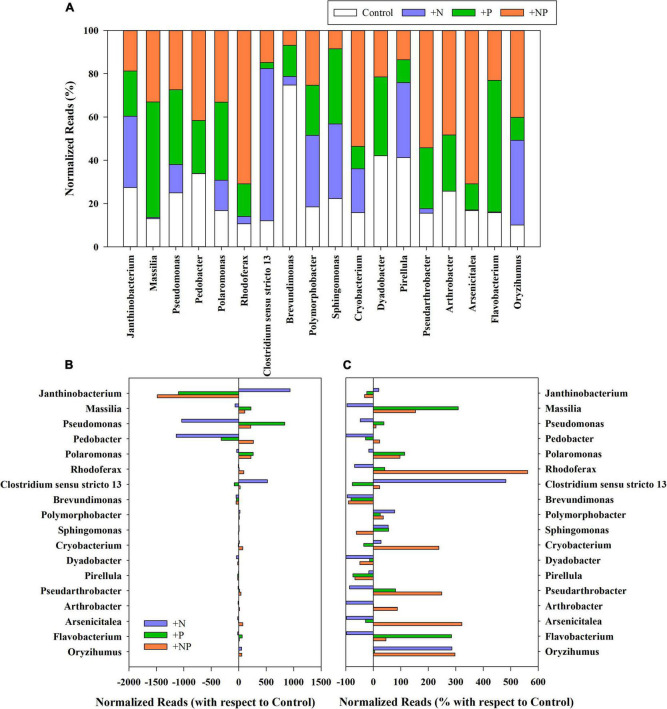
**(A)** Relative abundance (%) of rarefied reads of the main genera present in the different treatments (C, control; P, phosphorus; N, nitrogen; and NP, nitrogen and phosphorus). **(B)** Variation of the normalised reads in the different nutrient amendment treatments with respect to the control for the main genera present in the studied microbial mat. **(C)** Percentage of variation of the normalised reads in the different nutrient amendment treatments with respect to the control for the main genera present in the studied microbial mat. Only genera with more than 0.1% of the total reads in all treatments have been represented.

The experimental alteration of nutrient concentrations promoted some structural changes in the prokaryotic community (Figures 2, 3). Among the dominant phyla, Proteobacteria slightly varied (Figure 2A), with this phylum being, by far, the most abundant in the original mat, these changes were quite small in percentage compared to the controls without nutrient amendments (Figure 2B). Firmicutes showed huge increases compared to the undisturbed mat mostly when nitrogen (+N) was added, whereas the addition of phosphorus promoted small decreases. Both Actinobacteriota and Bacteroidota responded more favourably when both nutrients (+NP) were jointly supplied (Figure 2), although any single nutrient additions were associated with decreases in the abundance and relative importance of the latter. In relative terms, these shifts in the community were more important for Actinobacteriota and, particularly, for Firmicutes. Cyanobacteria appeared to be favoured by +N and +NP amendments when compared to the controls.

Variations of prevalent genera linked to the nutrient amendments in the different treatments (+N, +P, and +NP) relative to the undisturbed mat are shown in Figure 3. The net increase of Proteobacteria in the nitrogen-amended treatment was mainly attributed to the genus *Janthinobacterium*, which was already dominant in this mat, although this genus decreased in both treatments with phosphorus (P and +NP) amendments (Figures 3B,C). Other Proteobacteria, such as those of genus *Pseudomonas*, mainly increased when phosphorus (+P) was the amended nutrient. For Firmicutes, the huge increase in the treatment amended with nitrogen (+N) was mainly linked to a higher abundance of the genus *Clostridium*. On the contrary, the increase of Bacteroidota and Actinobacteriota phyla when both nutrients were jointly supplied (+NP) was mainly due to the increased abundance of *Pedobacter* and several members of the order Micrococcales (families Microbacteriaceae, Intrasporangiaceae, and Micrococcaceae), respectively. Cyanobacteria increased in relative terms in all treatments, but, more particularly, when nitrogen was added, either alone (+N) or with phosphorus (+NP). The highest increases were mainly shown by genera *Phormidium* and *Leptolyngbya* in the +N treatment.

### Restructuring of the phototrophic community after nutrient enrichment traced by taxa-specific pigments

**FIGURE 4:**
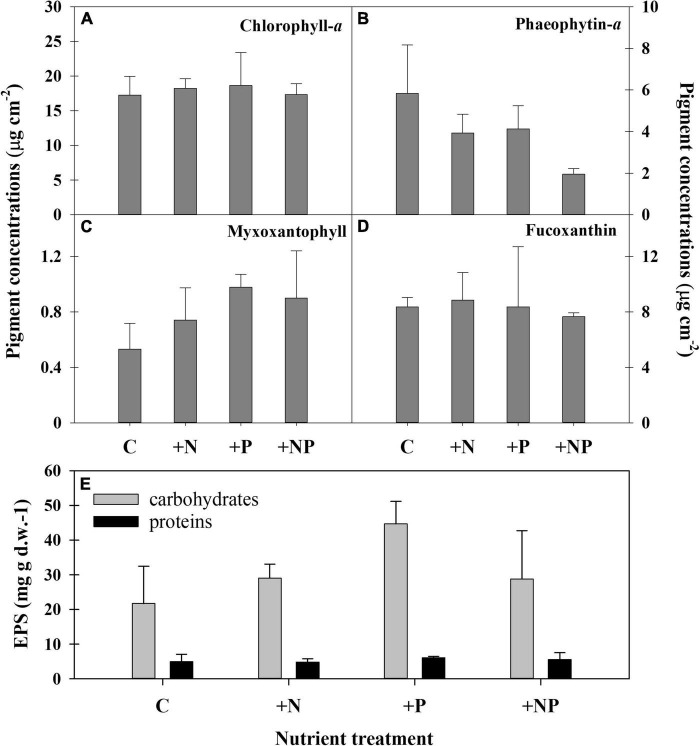
Pigment concentrations in the different experimental conditions at the end of the experiment. **(A)** Chlorophyll *a*, **(B)** phaeophytin-a, **(C)** myxoxantophyll (cyanobacteria biomarker), **(D)** fucoxanthin (diatom biomarker). C refers to the control treatment. **(E)** Content of two exopolymeric substances (EPS) fractions (carbohydrates and proteins) in the different treatments at the end of the experiment.

The ANOVA *post hoc* test for differences in taxa-specific biomarker pigments between the different experimental conditions is shown in Table 2, and the data are shown in Figure 4. The experimental enrichment resulted in no statistically significant effects on total photosynthetic biomass, measured as the areal chlorophyll *a* (Chl-a) content (Figure 4A). Even so, a statistically significant decrease of the phaeophytin-a, a Chl-a degradation product, was observed in the treatment +NP (Figure 4B), which would mean healthier populations. Also, both +N and +P treatments showed lower phaeophytin-a amount compared to controls, although this difference was not statistically significant. Concerning taxon-specific carotenoids, expressed as their ratio to Chl-a, phosphorus fertilisation, both alone (+P) and combined (+NP), was associated with an increase of the myxoxanthophyll (specific of cyanobacteria) vs. the Chl-a ratio (Figure 4C). This could mean a relative favouring of cyanobacteria compared to eukaryotic algae that also contain Chl-a, although differences compared to controls were only significant when P was added alone. On the contrary, in both treatments supplemented with phosphorus (+P and +NP), a slight, although no, statistically significant decrease of fucoxanthin (specific of diatoms) vs. the Chl-a ratio occurred (Figure 4D). This structural shift of the photosynthetic community was slightly reflected in the relationship between fucoxanthin and myxoxanthophyll, although this trend did not show statistical significance. Lower values of this ratio occurred at treatments involving fertilisation with phosphorus (+P and +NP), while the highest were registered in the controls. Contrastingly, with other microbial mats from the Byers Peninsula mainly formed by cyanobacteria, the photoprotective pigment scytonemin was not found in the studied mat, neither before nor after the treatments.

**TABLE 2:** The *post hoc* test (*p*-values) for differences in pigments, elemental composition, and EPS content between the different experimental conditions.

	C vs. +N	C vs. +P	C vs. +NP	+N vs. +P	+N vs. +NP	+P vs. +NP
Chl-a	n.s.	n.s.	n.s.	n.s.	n.s.	n.s.
Pheo-a/Chl-a	n.s.	n.s.	0.055	n.s.	0.042	0.036
Myxo/Chl-a	n.s.	0.022	0.111	n.s.	n.s.	n.s.
Fucox/Chl-a	n.s.	n.s.	n.s.	n.s.	n.s.	n.s.
Carbon	n.s.	n.s.	n.s.	n.s.	n.s.	n.s.
Nitrogen	n.s.	n.s.	n.s.	n.s.	n.s.	n.s.
Phosphorus	n.s.	n.s.	0.036	n.s.	n.s.	n.s.
C/N	n.s.	n.s.	n.s.	n.s.	n.s.	n.s.
C/P	n.s.	n.s.	n.s.	n.s.	n.s.	n.s.
N/P	n.s.	n.s.	n.s.	n.s.	n.s.	n.s.
EPS-carbohydrates	n.s.	0.049	n.s.	0.047	n.s.	n.s.
EPS-proteins	n.s.	n.s.	n.s.	n.s.	n.s.	n.s.

ns = *p* < 0.12.

### Nutrients stoichiometry and exopolymers (extracellular polysaccharides) content

No statistically significant differences could be demonstrated among the different treatments for the elemental composition (C-N-P) of the mat (except for the higher P content in the +NP treatments compared with the controls), as changes with experimental fertilisation were modest and displayed a high dispersion within groups (Table 2). The content of exopolymers (EPS) in the mats experimentally amended with nutrients was also quantified, being always more abundant in the fraction of carbohydrates compared to that of proteins (Figure 4). Among treatments, carbohydrates showed a statistically significant (Table 2 and Figure 4) increase in the treatment supplemented only with phosphorus (+P) compared to both the controls (C) and the treatments fertilised only with nitrogen (+N). No statistically significant differences among the treatments were observed in the protein EPS content.

### Autotrophic carbon metabolism: Trends in the vertical profiles of photosynthesis, oxygen evolution, and H^13^CO_3_ uptake

**FIGURE 5:**
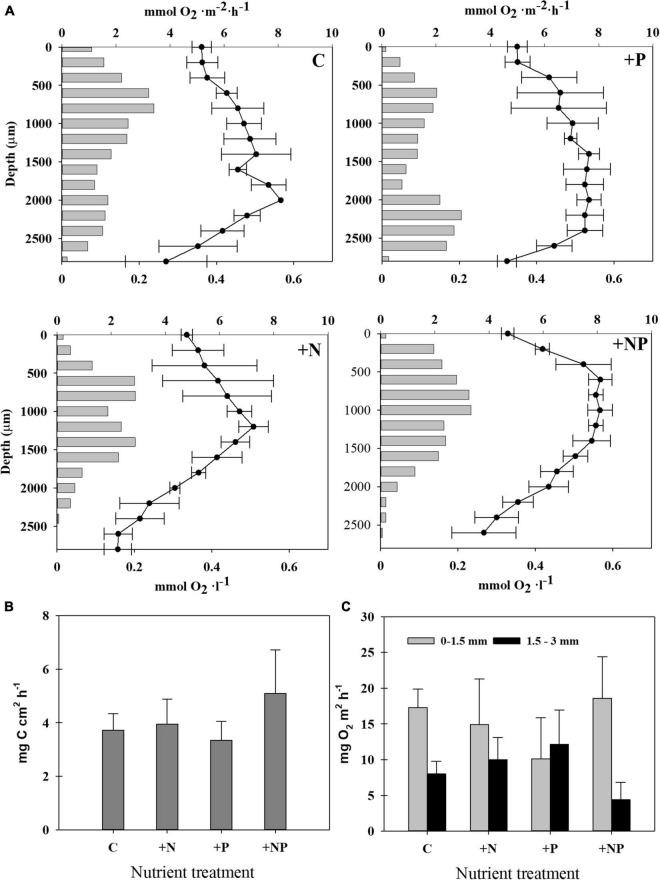
**(A)** Steady-state oxygen profiles (line, mmol O_2_ l^–1^, mean ± SD from three replicates) and rates of gross photosynthesis at each depth (bars, mmol O_2_ m^–2^ h^–1^, mean from three replicates) in the different treatments: Control (C): upper left; +N: upper right; +P: lower left; +NP: lower right. **(B)** Photosynthetic activity was measured as rates of inorganic carbon assimilation in the different treatments at the end of the experiment. Incubations were made under illuminated conditions (average PAR: 662-mol photons m^–2^ s^–1^). **(C)** Gross photosynthetic oxygen production rates were measured with microelectrodes in the different treatments at the end of the experiment. The two measures shown for each treatment are the result to integrate two different layers in the mat, 0–1.5 mm (grey) and 1.5–3 mm (black).

Photosynthetic activity of the mat was assessed before and during the experiment in the controls and nutrient amended mats using diverse approaches, namely, by measuring the incorporation of isotope tracers (NaH^13^CO_3_), as well as performing oxygen microprofiles (Figure 5). Up to five different C-photoassimilation measurements with ^13^C were conducted in January with natural mat samples without any additions. In these measurements, rates of inorganic carbon photoassimilation ranged from 4.19 ± 0.30 μg C cm^–2^ h^–1^ to 9.57 ± 3.10 μg C cm^–2^ h^–1^. This corresponds to 37.3 ± 2.7 to 109.8 ± 35.9 μg C g^–1^d.w. h^–1^, and to 90.8 ± 6.7 to 147.10 ± 48.22 μg C mg^–1^ chl-*a* h^–1^, respectively, the highest rates corresponding to sunny day incubations. The highest photosynthetic activity (c.a., 25%, 5.09 ± 1.62 μg C cm^–2^ h^–1^) measured by NaH^13^CO^3^ incorporation was detected in the microcosms simultaneously amended with both nitrogen and phosphorus (+NP) (Figure 5B). However, differences of this treatment with the others were not statistically significant (*p* > 0.05), given the relatively high dispersion of the data within each treatment.

Microelectrode studies allowed us to establish profiles of oxygenic photosynthesis along the vertical mat profile (Figure 5A). As a general pattern, mats from all types of conditions assayed showed two peaks of oxygenic photosynthesis, which coincided with the zones of dominance of diatoms (subsurface peak) and cyanobacteria (deep peak). The subsurface maximum was found at about 0.6 mm depth, except for the +NP treatments, where it was located at a depth of around 0.8 mm. Contrastingly, with the rest of the conditions, the deep photosynthetic peak was not so clear for the +NP treatment, and, if so, it was located at around 1.4 mm for the deep peak for N treatments. The deep photosynthetic maximum was deeper, however, for the controls (around 2.0 mm) and, especially, for the P treatments (located at around 2.2 mm depth). The depth of the euphotic layer, which is defined as the depth where the photosynthetic rates are still detectable, was also higher in the controls and +P treatment (around 3 mm) compared to both treatments involving nitrogen addition (up to 2.5 mm).

Given this vertical heterogeneity, two depth-integrated rates of gross photosynthetic were calculated by splitting the total profile into two zones, namely, from 0 to 1.5 mm and from 1.5 to 3 mm, respectively (Figure 5C). In all treatments except in that fertilised only with phosphorus, the photosynthetic rates were higher in the upper layer (0–1.5 mm). These differences were statistically significant in the controls (*p* = 0.007) and in the treatment +NP fertilized with both nutrients (*p* = 0.018).

### Nitrogen metabolism: uptake of inorganic forms and N_2_ fixation

**FIGURE 6:**
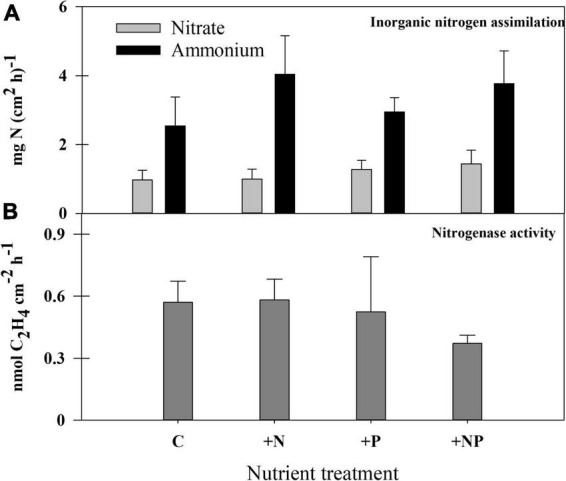
**(A)** Rates of inorganic nitrogen (nitrate and ammonium) assimilation and **(B)** nitrogenase activity in the different treatments at the end of the experiment.

The assimilation of inorganic nitrogen compounds (nitrate and ammonia) was measured by adding these compounds labelled with ^15^N (Figure 6A), both for undisturbed control mats and for the experimental microcosms. All these experiments confirmed that ammonium was a preferable nitrogen source compared to nitrate (*p* < 0.05), whose uptake rates were less than half of ammonium uptake rates. In the different experiments, nitrate uptake appears to be 300–500% higher in the light than in the dark, whereas ammonium uptake rates were also higher in illuminated samples, but only by 25–90%; all these differences were statistically significant (*p* < 0.05). No remarkable differences in nitrate uptake were found among the different conditions assayed, whereas, as a general rule, N-treatments (both alone and with phosphorus) showed the highest ammonium uptake rates than non-N-amended microcosms, although the standard deviation of results within each treatment was high, and these differences were not statistically significant (*p* > 0.05).

Nitrogen fixation (acetylene reduction activity – ARA) in the studied mat was almost negligible, both in unperturbed mats and in the amended experimental settings (similar results for all treatments), with actual measurements (around 0.3 nmol C_2_H_4_ cm^–2^ h^–1^) bordering the detection limit of the method. The method was contrasted with other microbial mats from the Byers Peninsula, which showed much higher rates (by several orders of magnitude) than the studied mat. Still, the combined addition of both nutrients caused a statistically significant (*p* = 0.0053) decrease in the rates of N_2_ fixation compared with the other treatments (Figure 6B).

### Changes in the functional metabolic prediction with PICRUSt2

The PICRUSt2 functional prediction based on the potential occurrence of selected genes (KO Functional orthologs) was used as a proxy for metabolic changes in the community as a response to the experimental nutrient enrichment. The main functions explored were those related to the uptake of major nutrients, autotrophy, exopolysaccharide biosynthesis, and stress response (Supplementary Table 1). With regard to the metabolisms of nitrogen, main increases were found in treatment +N compared to the undisturbed control for genes encoding the ferredoxin-nitrite reductase (nirA and nirB), which are involved in the assimilation of inorganic nitrogen. Proxies of denitrification and anammox metabolisms (nirK and nirS) also increased but, in this case, when both nutrients were jointly added (+NP). However, the gene encoding the nitrogenase iron protein (NifH), which mediates the N_2_ fixation, was predicted to decrease in the treatment fully supplemented with nutrients (+NP). With regard to the metabolism of phosphorus, an increase of gene-encoding high-affinity phosphate-specific transporters (e.g., pstA) was predicted to occur when only nitrogen (+N) was added without a balanced complement of phosphorus, similar to what was observed for components of the aerobic respiratory chain (cyoA). The highest increases in the proxy of photosynthetic metabolism (psbA gene) were also predicted for the +N treatment. However, genes related to the biosynthesis of exopolysaccharide showed higher increases when phosphorus was added (+P, CpsB gene). In the treatment supplemented with phosphorus (+P), regulators of stress factors (RsbU gene) were also predicted to increase.

### Relationships between environmental variables, prokaryotic community structure, and predicted metabolic functions in the experiment (distance-based redundancy analysis)

**FIGURE 7:**
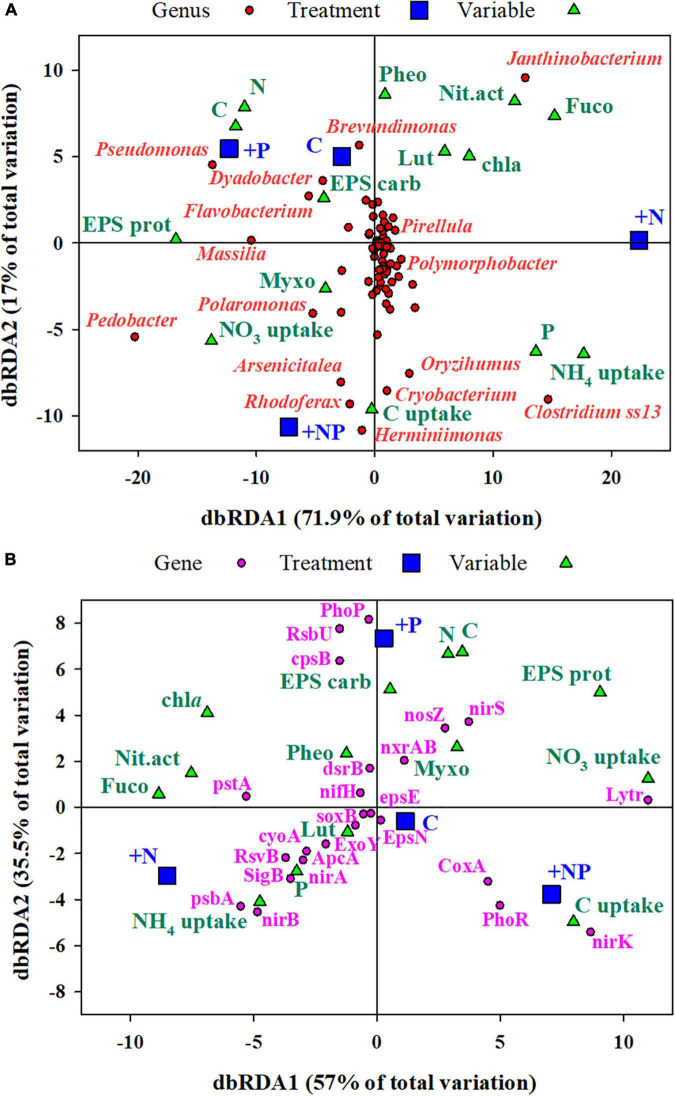
**(A)** Distance-based redundancy analysis (db-RDA) of **(A)** all the ZOTUs (grouped at the taxonomic level of genus) and **(B)** selected genes (Supplementary Table 1 for description) from Picrust2, analyses of 16SrRNA amplicon sequencing. Predictor variables for both dbRDA were C, carbon; N, nitrogen; P, EPS carb, EPS carbohydrates; EPS prot, EPS protein; chla, chlorophyll *a*; Myxo, myxoxanthophyll; Fuco, fucoxanthin; Lut, lutein; Pheo, pheophytin; C uptake, NO_3_ uptake, NH_4_ uptake, and Nit.act, nitrogenase activity. Genera with more than 0.1% of the total reads in all treatments have been shown.

A first distance-based redundancy analysis (db-RDA) was carried out with all the taxonomic units using biochemical and metabolic parameters measured in the mat as explanatory variables (Figure 7A). The first axis accounted for 71.9% of the variance and separated in its positive side the +N treatment from the others. The main families of prokaryotes associated with this +N treatment were *Janthinobacterium* and *Clostridium*. The main explanatory variables related with this treatment were the uptake of NH_4_, nitrogenase activity, and the content of Fucoxanthin (diatoms proxy). On the opposite side of the axis, the abundant taxa mainly associated with treatments C (undisturbed mat) and +P were *Pseudomonas*, *Flavobacterium*, and *Massilia*, whereas *Polaromonas* was more related to the +NP treatment. The second axis explained 17% of the variance with the C and +P treatments located on its positive side.

A second dbRDA was performed based on the prediction (based on taxonomy) of genes associated with the metabolism of major nutrients, stress, autotrophy, and exopolysaccharide biosynthesis (Figure 7B). Specific genes used in the analysis and their functional role are listed in Supplementary Table 1. The same predictor variables as in the previous db-RDA were used here. The first axis (dbRDA1) explained 57% of the total variation and, similarly, separated the +N treatment, now in the negative side, from the others. The main genes associated with the +N treatment were those encoding for the high-affinity phosphate-specific transporter (pstA), proteins of the photosynthetic system (psbA), major components of the aerobic respiratory chain (cyoA), and the nitrate assimilation operon (nirA and nirB). On the contrary, proxies of denitrification (nirK and nirS) and a gene encoding for biofilm regulatory proteins (lytR) showed the highest loadings in the opposite side of this axis, in association with the other treatments, particularly with +NP. The second axis, which explained 35.5% of the variance, mainly separated the +P treatment from the others, with genes related to encoding for one of the regulators of the phosphorus uptake system (PhoP), with genes required for exopolysaccharide production (CPsB), and genes related to the regulation of stress factors (RsbU).

## Discussion

The present study is the first attempt to study the effects of inorganic nutrient additions on the structure and function of a maritime Antarctic microbial mat. Similar experimental approaches have been only occasionally assayed in polar regions (Bonilla et al., 2005; Kohler et al., 2016). In our case, the ambient concentrations of nutrients show a marked gradient between the overlying and interstitial waters, which agree with the observed in other polar microbial mats, both in Antarctica (Vincent et al., 1993) and in the Arctic (Mueller and Vincent, 2006). It likely implies a higher nutrient availability for mat microorganisms than that expected when referring to the concentrations in the stream waters. Besides, not only the amounts of nutrients but also their stoichiometry differs between overlying and interstitial waters. Although, as indicated by Bonilla et al. (2005), it does not imply necessarily the lack of nutrient limitation, because the higher biomass packaging in the mat requires a higher amount of nutrients for sustained growth. Although the nutrient concentrations used in the nutrient-amended microcosms were far higher than those in the water overlying microbial mats, the relatively high concentrations of both inorganic nitrogen and phosphorus measured in the interstitial water within the sediment may partly explain, together with other factors, the relatively small response of the autotrophic microorganisms from this microbial mat to increased levels in inorganic nutrients of overlying waters. These chemical gradients occur in the interface of sediment and water.

Our experimental manipulation did not generate a detectable accretion of the mat; still, some structural and functional changes occur. Although the total algal biomass remains (statistically) unchanged, a decline in the phaeophytin-a content was found in all treatments, entailing any type of fertilisation, whatever nitrogen or phosphorus, particularly if both nutrients were added in combination. Phaeophytin-a is a degradation form of chlorophyll *a* that is produced when the magnesium linked to the aromatic ring of the molecule is detached. The relative rise of this derivative can be interpreted as the increase in the senescence processes of algal populations (Rochera et al., 2013). In our study, it seems then that the balanced availability of nutrients improves primary production, although a net increase of biomass could not be unequivocally demonstrated. Furthermore, the relative decrease of phaeophytin-a is less marked in those treatments merely supplemented with one nutrient (+N and +P). It indicates that only a stoichiometrically balanced addition of both nutrients can fit the requirements of the phototrophic community, probably by the fact that a single addition of one nutrient would induce limitation by the other, once the non-amended nutrient pool is exhausted. This drop in the phaeophytin-a/Chl-a quotient has been also observed in a similar experiment and has been interpreted in similar terms of healthier mats, showing a lower phaeophytin-a to the Chl-a ratio (Camacho and de Wit, 2003).

Both the combined (+NP) and the single (+P) fertilisation with phosphorus caused a weakening of the mat aggregation, which is evident by the fluffy consistency that showed the mat in these cases. It is possible that the higher oxygen evolution produced by the increase of the photosynthesis created an excess of gas bubbles, which caused parts of the mat to lift off. In part, this idea is sustained by the observed by Shepard and Sumner (2010), who studied the influence of the light intensity in the growth of biofilms. As noted by these authors, the gas bubbles generated during photosynthesis become more abundant when irradiance increases, thus influencing greatly the morphology of a mat. By contrast, they conjecture that a slower growth should result in more organised patterns, which seems to be the opposite of the observed in our mat. Thus, other possible explanations, such as those derived from the changes in the structure and functioning of the microbial community, are explored below.

The fertilisation with phosphorus promoted in our case further growth of non-heterocystous cyanobacteria relative to diatoms, which is deduced by the increase of myxoxanthophyll relative to fucoxanthin. Interestingly, this increase is higher when phosphorus is added alone rather than when it is added jointly with nitrogen. Also, the single addition of phosphorus favoured the development of cyanobacteria over diatoms in similar studies (Camacho and de Wit, 2003), thus suggesting that cyanobacteria could be less competitive if the availability of phosphorus is limited. However, as commented above, it is the phosphorus addition rather than the combined supply with nitrogen that induces the higher effects. Probably, the pool of nitrogen existing inside the cells, or its recycling inside the mat, is sufficient for covering the cyanobacterial demands.

These directional changes observed in the structure of the phototrophic community according to changes in the ratio of the resource availability would agree with the predictions by Tilman’s resource-ratio hypothesis, as observed in numerous studies (Miller et al., 2005). This hypothesis assumes that each species would outcompete others under a particular proportion (ratio) of the limiting resources. Accordingly, the relative variation of a resource in our experiment, although its total amount remains unchanged, may produce a selective pressure that causes a redistribution of species dominance. Furthermore, it seems that a counterbalanced redistribution of the biomass of cyanobacteria and diatoms occurs, although this does not produce a perceptible accretion of the mat. A competitive advantage of cyanobacteria over diatoms may respond to different mechanisms. When nitrogen is in short supply relative to phosphorus, non-nitrogen-fixing cyanobacteria can obtain nitrogen from reserve polymers, such as phycobiliproteins or cyanophycin (Watzer and Forchhammer, 2018). Also, different from diatoms, cyanobacteria can alleviate the nitrogen requirements from the N_2_ fixation. In our case, rates of ethylene production are the lowest in the treatment fertilised with both nutrients, which suggests some regulation of the nitrogenase activity, depending on the relative quota of nitrogen and phosphorus. Anyhow, the abundance of heterocystous cyanobacteria is negligible in this mat, although they might acquire a higher relevance if the availability of nutrients changes significantly as they appear in mats nearby (Fernández-Valiente et al., 2007). Therefore, in accordance with the outcomes of our experiment, it is conceivable that the assembling patterns observed in the structure of phototrophic benthic communities from Byers (Fernández-Valiente et al., 2007) when they are active during the Antarctic summer period may be related, at least in part, to what is predicted by Tilman’s resource-ratio hypothesis.

Our inorganic carbon uptake results agree with the extended idea that high primary production but also low growth rates are both inherent attributes of microbial mats. Even though the combined addition of nutrients produced an increase of photosynthetic activity, this was not translated to a substantial increase in the carbon content. Similarly, neither the nitrogen content in the fertilised mats appears to be altered substantially by fertilisation. By contrast, all the treatments involving fertilisation show somewhat higher accumulation of phosphorus, thus producing a drop in the C/P and N/P molar ratios. Low ratios between the carbon and other major nutrients have been attributed to a higher capacity of these benthic communities to adsorb and immobilise nutrients (Cross et al., 2005). However, we were unable to explain why the accumulation of phosphorus also occurs when the mat is supplied only with nitrogen, although, anyhow, these differences were not statistically significant.

Our study with microelectrodes shows that primary production in the mat is allocated differently, depending on the nutritional conditions. Particularly, when only phosphorus is added, the development of a deeper photosynthetic maximum is more evident. Also, this treatment shows a lower chlorophyll *a* content relative to carbon. This may occur if an important part of carbon is devoted to the synthesis of EPS, whose amount is the highest in the +P treatment. An important part of these EPS might derive from the cells sheaths, which seems reasonable since cyanobacterial growth is improved in this treatment, as well as from heterotrophic bacteria that are known to produce high amounts of EPS in natural environments, such as those of the genus *Pseudomonas*. Besides, and following the idea of Decho et al. (2003), the EPS matrix may indirectly explain the deepening of the photosynthetic maximum observed in the treatment supplied with phosphorus. In the study carried out by these authors with sediments from the Bahamas, they concluded that the EPS matrix, when occurring in sufficient amounts, might reduce the spectral reflectance of the surface. This reduction enhances the forward scattering of light and consequently increases its penetration into the sediment. A direct consequence would be the deepening of the euphotic layer inside the mat.

Another possibility is the occurrence of a cross-feeding between the production of EPS and bacterial respiration that would explain the higher downward fluxes of oxygen observed at the deepest layers. Photosynthetic activity inside the mat, favoured by the limited diffusion, improves oxygen evolution and increases the pH due to CO_2_ consumption. Under these superoxic and alkaline conditions, the production of glycolate and other photosynthates deriving from short-chained carbohydrates can be enhanced (Bateson and Ward, 1988). A study by Nold and Ward (1996) exploring the nature of metabolites produced during the photosynthetic activity in a thermophilic cyanobacterial mat indicated that the photosynthate partitioning accumulated mostly polyglucose (up to 70% of total incorporated carbon). Interestingly, the accretion of the mat in this case was also limited, as the carbon deserved for the synthesis of macromolecules associated with growth (i.e., protein and rRNA) was considerably low. These carbohydrates can be readily incorporated by heterotrophic bacteria, which avoid the carbon flushing out from the mat. This is consistent with the idea that both a high metabolic activity and, also, slow accretion are inherent characteristics of these microbial communities.

The NH_4_ uptake exceeds notably the rates of NO_3_ assimilation in all treatments as well as in the controls, which is expected, considering that NH_4_ has been largely considered the preferred form of N for algal uptake, as being energetically more favourable to be directly used in this reduced form similar to the organic N in the amine group (Glibert et al., 2016). It is known that high ambient concentrations of NH_4_ may inhibit strongly the uptake of NO_3_ by a competence for the solute in photosynthetic organisms (Dortch, 1990). However, as observed for carbon, no significant increase in the nitrogen content occurs in the mat. This could be related to the increase of the dissolved organic nitrogen (DON) concentrations observed in the overlying water of both treatments involving nitrogen fertilisation (+N and +NP). Significant concentrations of DON have also been measured in the pore water of microbial mats from the McMurdo Dry Valleys (Antarctica) and have been related to the biological transformation of nitrogen in the mat (Sohm et al., 2020). Again, it would mean that transformation but not a significant net incorporation of nitrogen occurs in the studied mat, which underscores the idea that this community could be near to a steady equilibrium state.

All samples of the enrichment mat experiment were primarily subjected to microscopic morphological analysis of their cyanobacterial components before going into molecular studies. All of the morphotypes identified here have been previously cited in microbial mats from this area. *Phormidium* is one of the most frequently reported taxa, usually found in streaming waters, comprising a sort of morphotypes and ecotypes (Strunecký et al., 2012). *Leptolyngbya*, which forms the matrix of the mat, is a dominant species of finely laminated, prostate and lift-off mats even in deeper continental lakes (Sabbe et al., 2004). This cyanobacterium was also reported to form the matrix of the Casten Pond mat from the McMurdo Ice Shelf ablation zone close to Bratina Island (De Los Ríos et al., 2004). Non-heterocystous filamentous taxa were observed neither in the morphological nor in the molecular analyses. This is a significant feature of benthic cyanobacteria of maritime Antarctic lakes and ponds (Ellis-Evans, 1996). Even in Antarctic continental lakes, *Nostoc* spp. appears to be the only nitrogen fixer, and the process is the restricted to the lake shores (Allnutt et al., 1981). Anyhow, the possibility of nitrogen fixation by non-heterocystous cyanobacteria cannot be excluded (Zehr et al., 2001). This could be supported by the presence of *Chroococcidiopsis* sp. where the abundance of filamentous cyanobacterial forms was lower. *Chroococcidiopsis* was described to dominate in extreme habitats, including sites of the Antarctic continent where ice melts for at least 2 months a year (Vincent, 2000). Different isolates of this species were found to fix the nitrogen but only when grown in microaerobic conditions (Boison et al., 2004). Anyhow, and even with the relatively low nitrogen fixation rates recorded in all treatments and the control, nitrogen fixation could be performed not only by cyanobacteria but also by other non-phototrophic bacteria, for which key genes for this activity (e.g., *nifH*) have been found within the bacterial assemblages of the studied mat. Major examples of these non-phototrophic bacteria found in the studied mat for which PICRUSt2 also predicts the occurrence of the nifH gene are genera *Paenibacillus*, *Acetobacterium*, and *Desulfosporosinus*, as well as diverse members of the *Rhodobacteraceae* family.

Post-nutrient treatment changes were also found for the structure of the heterotrophic prokaryotic community. The highest favourable response to the addition of N was found for *Janthinobacterium*, a typical genus from cold environments (Rassner et al., 2016). This bacterium can conduct simultaneously heterotrophic nitrification and aerobic denitrification (Chen et al., 2019), particularly when N is highly available with respect to C as in this treatment. Firmicutes also benefited when N was sufficiently available, which corresponds with the idea that this phylum is a key player in ammonium assimilation metabolism and nitrogen cycling (Amon et al., 2010). These observations at the taxonomic level, suggesting an enhancement of the microbial nitrogen metabolism, agree with PICRUSt2 results, which predict an increase of genes encoding the nitrite reductase (nirA) and other associated proteins (nirB). The gene encoding for the phosphate membrane transporter protein (pstA) is also predicted to increase in the treatment amended only with N. This permease protein is part of the high-affinity phosphate uptake system, which is induced when the concentration of inorganic phosphate in the environment is low or unbalanced as in the N-amended treatment.

On the other hand, our experiment demonstrates how an increase in the availability of P may produce a shift toward a heterotrophic community able to use or metabolising phosphate. *Massalia*, a phylogenetically related genera to *Janthinobacterium*, for instance, showed a high positive response when P was added, either alone or with N. Species of this genus have the ability to solubilise diverse phosphate sources in soils (Zheng et al., 2017). They can be then considered as phosphate-solubilising bacteria, which represent a metabolic advantage when P solubilisation is hindered. Accordingly, differential development of *Massilia* has been observed in routine dosage of P in drinking water to avoid corrosion (Douterelo et al., 2020). Thus, the P fertilisation conducted in the experiment should make it, at first, available to the entire community. However, a substantial part of this P is expected to be progressively precipitated after application, and the consequence is that this would become a selective force driven by microorganisms competition. Other bacterial genus favoured by the supply of phosphorus in the experiment is *Pseudomonas*. This has been described as a relevant producer of polyhydroxyalkanoates (PHAs) in freshwater ecosystems within maritime Antarctica (Ciesielski et al., 2014), which represents a strategy for carbon storage under the stress of nutrient limitation, especially when this is affecting the availability of phosphorus.

These directional changes in the community are also predicted at the functional level by the PICRUSt2 analysis. This predicts an increase of the nitrate assimilation metabolism, as commented previously, in response to nitrogen fertilisation. Equally, when nitrogen is not provided with a balanced addition of phosphorus, as it occurs in the treatment +N, an increase of genes-encoding high-affinity phosphate-specific transporters (e.g., pstA) is predicted. Under limited phosphorus availability, as in the +N treatment, bacteria with these genetic mechanisms can switch on the synthesis of this phosphate transport system (Hudek et al., 2016), so it is expected that components of the community holding this system are favoured under these conditions. However, the predicted increase of genes involved in the production of exopolysaccharide (i.e., CpsB) would be related to the increase in the EPS production observed in the nitrogen-amended treatments (both +N and +NP) compared to the controls, although this increase is lower than that in the +P treatment.

## Concluding remarks

In summary, this study improves our knowledge of the functioning of microbial mats in Antarctic ecosystems. Our findings also provide additional confirmation of Tilman’s resource-ratio hypothesis (Tilman, 1982; Tilman et al., 1986) in a physically constrained environment, thus demonstrating that different biological groups are specialised in different nutrient ratios. The results showed both assimilation and release processes to be closely balanced, which results in low mat accretion. The mat exhibits a slight response to the nutrient amendments, but, still, it causes some functional and structural alterations. Extending the time of incubation would likely provide a more significant community (changes in abundance and evenness) and functional effects. With all, our results show convincing pieces of evidence that, besides other factors, a shift in the regional dynamic of nutrients might alter the metabolic equilibrium of these microbial communities. In this sense, the slight short-term changes observed in the species composition after fertilisation may ensue in important shifts with time. Therefore, although the major impact we showed took place at the physiological level for the first time, long-lasting effects could lead also to deep changes in the community composition. Considering the ubiquity of these mats in the area and other polar territories, they would have the potential to impact significantly on the whole carbon cycle of polar areas. The potential role of nutrients and their ratios in determining the mat structural and functional features unveiled by our study shows how physical restrictions, such as the low temperatures, the extended ice-cover periods, and the reduced irradiance, are not the only factors regulating Antarctic microbial communities, at least not during the summer in these less-extreme Antarctic areas. Accordingly, other abiotic factors and biotic interactions, such as the performance under different nutrient scenarios, may be equally important for the highly relevant microbial polar communities.

## Data availability statement

The data presented in the study are deposited in the NCBI repository, accession number: PRJNA817827.

## Author contributions

AC and CR conceived and designed the study. AC carried out the field experimental study and the physiological measurements, wrote the manuscript, and provided the funds for the study. CR conducted the biochemical analyses. AP conducted the molecular and bioinformatic analyses. CR and AP performed the statistical analyses and wrote some sections of the manuscript. All authors contributed to manuscript revision, read, and approved the submitted version.

## Footnotes

1
https://www.kegg.jp/
