# Supplementary material for: Effect of experimentally increased nutrient availability on the structure, metabolic activities, and potential microbial functions of a maritime Antarctic microbial mat
Source: Front Microbiol. 2022 Sep 23;13:900158. doi: 10.3389/fmicb.2022.900158 (PMC9539743; doi:10.3389/fmicb.2022.900158)
Supplement: Supplementary file 1 [file Table_1.DOCX]

**Supplementary Table 1.** Main genes selected from the Picrust2 analysis for the main metabolisms studied in the microbial mat.

| **KO** | **Gene** | **Cycle** | **Metabolic function** | **Description** |
| --- | --- | --- | --- | --- |
| K02588 | nifH | N | Nitrogen fixation | Nitrogenase iron protein nifh |
| K00376 | nosZ | N | Denitrification | Nitrous-oxide reductase |
| K00366 | nirA | N | Assimilatory Nitrogen Reduction | Ferredoxin-nitrite reductase |
| K00367 | nirB | N | Dissimilatory Nitrogen Reduction | Ferredoxin-nitrate reductase |
| K15864 | nirS | N | Annamox | Nitrite reductase (NO-forming) / hydroxylamine reductase |
| K00368 | nirK | N | Annamox/ Denitrification | Nitrite reductase (NO-forming) |
| K00370 | nxrAB | N | Nitrification | Nitrate reductase / nitrite oxidoreductase, alpha subunit |
| K07660 | PhoP | P | low Affinity P | Response regulator phop |
| K07636 | Phor | P | low Affinity P | Phosphate regulon sensor histidine kinase phor |
| K02038 | PstA | P | low Affinity P | Phosphate transport system permease protein |
| K02689 | psbA | C | autotrophy | Photosystem I P700 chlorophyll a apoprotein A1 |
| K02092 | APCa | C N | autotrophy | Allophycocyanin alpha subunit |
| K03090 | SigB |  | SigmaB stress | RNA polymerase sigma-B factor |
| K07315 | RsbU |  | SigmaB stress | Phosphoserine phosphatase rsbu/P |
| K04749 | RsbV |  | SigmaB stress | Anti-sigma B factor antagonist |
| K11181 | DSRb | S | Dissimilatory sulfate reduction | Dissimilatory sulfite reductase beta subunit |
| K17224 | SoxB |  | Thiosulfate oxidation by SOX complex | Sulfur-oxidizing protein soxb |
| K19423 | epsE |  | Exopolysaccharide Biosynthesis | Glycosyltransferase epse |
| K19430 | epsN |  | Exopolysaccharide Biosynthesis | Pyridoxal phosphate-dependent aminotransferase epsn |
| K07705 | Lytr | C | Exopolysaccharide Biosynthesis | Response regulator lytt |
| K00971 | CpsB | C | Exopolysaccharide Biosynthesis | Mannose-1-phosphate guanylyltransferase |
| K16566 | exoY | C | Exopolysaccharide Biosynthesis | Exopolysaccharide production protein exoy |
| K02274 | coxA | C | quimiotrophy | Cytochrome c oxidase subunit I |
| K02297 | cyoA | C | quimiotrophy | Cytochrome o ubiquinol oxidase subunit II |
